# Matrix-Assisted Plasma Atomization Emission Spectrometry for Surface Sampling Elemental Analysis

**DOI:** 10.1038/srep19417

**Published:** 2016-01-14

**Authors:** Xin Yuan, Xuefang Zhan, Xuemei Li, Zhongjun Zhao, Yixiang Duan

**Affiliations:** 1 Research Center of Analytical Instrumentation, College of Chemistry, Sichuan University, Chengdu, China, 610064; 2 Research Center of Analytical Instrumentation, Key Laboratory of Bio-resource and Eco-environment, Ministry of Education, College of Life Science, Sichuan University, Chengdu, China, 610064

## Abstract

An innovative technology has been developed involving a simple and sensitive optical spectrometric method termed matrix-assisted plasma atomization emission spectrometry (MAPAES) for surface sampling elemental analysis using a piece of filter paper (FP) for sample introduction. MAPAES was carried out by direct interaction of the plasma tail plume with the matrix surface. The FP absorbs energy from the plasma source and releases combustion heating to the analytes originally present on its surface, thus to promote the atomization and excitation process. The matrix-assisted plasma atomization excitation phenomenon was observed for multiple elements. The FP matrix served as the partial energy producer and also the sample substrate to adsorb sample solution. Qualitative and quantitative determinations of metal ions were achieved by atomic emission measurements for elements Ba, Cu, Eu, In, Mn, Ni, Rh and Y. The detection limits were down to pg level with linear correlation coefficients better than 0.99. The proposed MAPAES provides a new way for atomic spectrometry which offers advantages of fast analysis speed, little sample consumption, less sample pretreatment, small size, and cost-effective.

In plasma based atomic spectrometry, the sample introduction has long been considered as the Achilles’ heel^1^. The development and improvement of sample introduction systems have been one of the main challenges for scientists working in this field^2^. Conventional pneumatic nebulization as the most common sample introduction device suffers from low nebulization efficiency (<5%), high sample uptake rates (1–2 mL min^-1^) and serious matrix interferences, which is problematic when sample volumes are small or sample with complex matrices^3^,^4^. Later technologies such as micro-nebulizers and electrothermal vaporization (ETV) providing higher sample introduction efficiency with reduced sample consumption have indeed attracted some interests^5^,^6^,^7^. However, these technologies still have some shortcomings, such as expensive equipment required in nebulizer with the increased risk of clogging^8^,^9^ and serious memory effects on ETV^10^. In recent years, electrolyte-cathode discharge (ELCAD) has proven to be successful for solution analysis such as solution-cathode glow discharge (SCGD) developed by Hieftje *et al*.^11^,^12^,^13^,^14^ and liquid-film dielectric barrier discharge (LFDBD) developed by Zhu *et al*.^15^. These liquid discharge devices offer advantages of small size, elimination the use of a nebulizer, low sample/power consumption, and cost effectiveness. However, most of the ELCAD are operated with flowing solution as one electrode, which necessitates the use of a sampling pump in the system^13^. Moreover, a known behavior about ELCAD is that the elemental emission intensity strongly depends on the pH of the solution, which restricted their applications^11^,^16^.

In the year 2004, Cooks *et al*. developed a new ambient surface sampling/ionization technique for mass spectrometry, termed desorption electrospray ionization (DESI)^17^. In the DESI technique, a pneumatically assisted electrospray jet is directed toward the sample surface to facilitate desorption/ionization. The DESI technique has several outstanding advantages over traditional techniques, including ambient sampling, high analysis speed, and direct sample analysis with little need for sample preparation/preseparation. Under the tide of the new scientific revolution, a mount of plasma-based techniques have been developed for ambient ionization methods^18^,^19^,^20^,^21^,^22^,^23^,^24^,^25^. Nevertheless, all of these techniques focus on the analysis of organic compounds with mass spectrometry. Few works have been reported in atomic spectrometry with plasma based surface sampling techniques. This is mainly due to the fact that the excitation energy and gas temperature of the plasma tail plume was too low to excite the atomic spectral information.

In chemical analysis, the introduction of sample matrix sometimes can bring unexpected effects. A typical example is the matrix-assisted laser desorption ionization mass spectrometry (MALDI/MS)^26^,^27^, which was developed for the analysis of molecules with large molecular weights. To obtain a signal, the analyte is mixed with a matrix compound. Most of the laser energy is absorbed by the matrix and then “transferred” to the analyte, which can be eventually ionized by the process. Therefore, the matrix is one of the most important factors in the success of the laser desorption technique. Recently, a matrix assisted ionization vacuum (MAIV) method was also developed. Compared to MALDI, laser is not required in vacuum of MAIV for ion formation^28^,^29^.

In this work, matrix assistance was utilized to couple with plasma atomization emission spectrometry for surface sampling for the first time. An appropriate matrix candidate should provide analyte auxiliary energy for atomization and excitation, and be absorbable to liquid analytes. Since filter paper (FP) is extremely simple and has already been successfully used in sample introduction, such as paper spray with DESI and MAIV^30^,^31^,^32^, it has been selected in this work as surface sampling matrix. This new method, termed matrix-assisted plasma atomization emission spectrometry (MAPAES), is simple in concept and instrument design, and is also easy for system operation. In most cases, MAPAES allows analytes present at matrix surfaces to be analyzed without any sample pretreatment, which simplified the analytical process. Furthermore, this method enables fast analysis with little sample consumption (sampling at 1-μL level, less than 1 min per sample). In addition, the detection can be accomplished through optical emission, which enables simultaneous detection of multiple elements. These advantages make the proposed MAPAES attractive as a potential miniaturized AES system for *in situ* and high-throughput elemental analysis.

## Results

### The optical emission process of MAPAES

**Figure 1:**
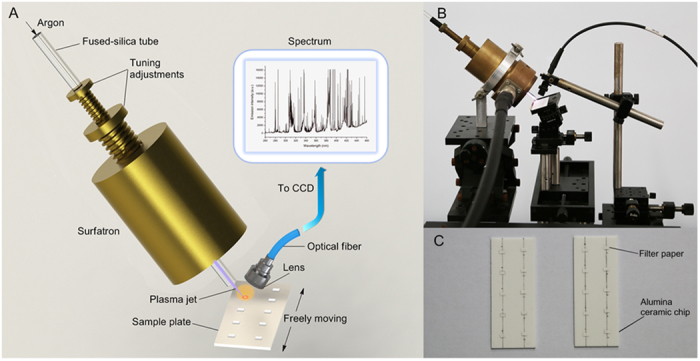
(**a**) Schematic diagram of the MAPAES system, (**b**) photographs of the experimental apparatus, and (**c**) sample plates.

The schematic diagram and photograph of the experimental setup are shown in Fig. 1(a,b) respectively. The tail plume of the home-built microwave-induced plasma jet was directly in contact with the ceramic sample plate surface at an angle of ~60°, forming an elliptic plasma region on the surface. Due to the thermal effect of the plasma, the sample solution adsorbed in the FP quickly evaporated even before touched by the plasma tail plume. Therefore, liquid samples were allowed to be detected immediately after being spotted on the FP without waiting for the FP to dry. After a few seconds treatment with plasma tail plume, the FP substrate was carbonized with its color turned black. To ensure an approximately same degree of carbonization, the carbonizing position was kept at about 2 mm away from the visible plasma region and the duration was fixed for 8 s. When the carbonized FP substrate being ablated by the plasma tail plume, it was instantaneously overheated and blazed up, which induced the sample atom released from the substrate, and the released sample atoms were simultaneously excited by the plasma in the ablation region. The ablation process was less than 2 s. The measurement time is about 15 s and the whole analysis process is within 1 min for a single sample.

The carbonization process was essential. Compared with noncarbonized fresh FP, the carbonized FP combusted much more quickly during ablation, which accelerated the sample atomization/excitation process, thus producing more intense and narrower pulse optical signals. In addition, combustion of carbonized FP emitted less visible light, which partly reduced the elevation of the spectral baseline in the visible region. Furthermore, after carbonization, the small piece of paper (2.5 mm × 1.5 mm, L × W) can be completely burned out one-time as a whole, which minimized the nonuniformity of combustion and fluctuation of signals caused by manual operation, and therefore helped to improve the reproducibility of the method.

For quantitative analysis, the size of filter paper is important. As peak height was used as analytical signal in this work, the whole filter paper must completely burn out in one time to give a single pulse signal. Therefore the filter paper must be limited in a small size. However, the absorptive capacity of filter paper decreases with its size reduction. It is also difficult to detect sample with very small size of filter paper, particularly for samples in low concentrations. Therefore, a compromised filter paper size of 2.5 mm × 1.5 mm, which can effectively absorb 1 μL liquid analyte and can be completely burned out in one time, was selected.

### Effect of FP sample matrix

**Figure 2:**
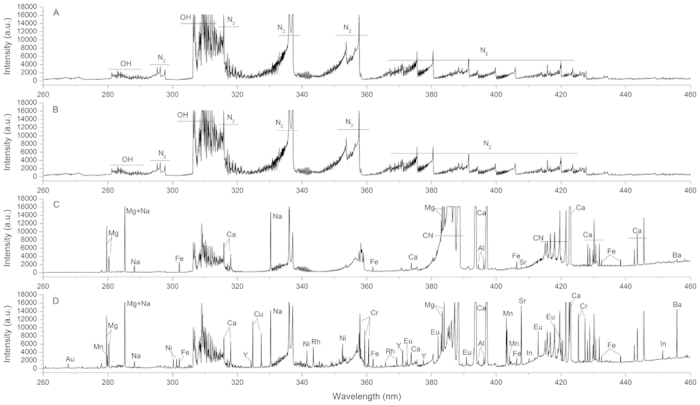
Emission spectra of a blank solution (5% HNO_3_) and solution spiked with an 11-element standard (Ba, Sr at 0.8 μg mL^−1^ and Au, Cr, Cu, Eu, In, Mn, Ni, Rh, Y at 4 μg mL^−1^) on ceramic and filter paper substrates. (**a**) Blank emission on the ceramic substrate; (**b**) emission spectrum of the 11-element standard on ceramic substrate; (**c**) Blank emission on the filter paper substrate; (**d**) emission spectrum of the 11-element standard on filter paper substrate.

To test the effect of FP sample matrix in the atomization emission process, two kinds of sample plates were used for comparison — one was ceramic chip baseplate with precut FP pasted on it for sample adsorption, the other was clean ceramic chip itself as the sample substrate. Blank solution (5% HNO_3_) and the solution spiked with an 11-elements standard (4 μg mL^−1^ of Au, Cr, Cu, Eu, In, Mn, Ni, Rh, Y and 0.8 μg mL^−1^ of Ba, Sr) were introduced onto both of the two substrates respectively: 3 μL on the clean ceramic chip surface, while 1 μL on the FP substrate. Due to the low absorbency of the slippy ceramic surface for liquid solution, air drying for about 2 h was performed only when clean ceramic chip was used as the substrate to prevent sample diffusion by plasma gas flow. The blank/samples spotted on the ceramic substrates were first ablated by the plasma tail plume, and then the FP substrates, so that the difference of the spectra can be distinguished easily. Figure 2 shows the typical emission spectra over the wavelength region between 260 and 460 nm. When the plasma tail plume touched the sample spot on the ceramic chip, no atomic spectral lines belong to analytes could be detected on the spectrum (Fig. 2(b)). The emission spectrum in Fig. 2(b) is almost the same as the background emission (Fig. 2(a)), with only several molecular emissions attributed to OH bands (band heads at 281 and 306 nm), and the N_2_ second positive system (band heads at 337, 357, 380, and 405 nm) found, indicating that the analytes were not excited by the plasma tail plume. The emission spectrum of ablating the clean FP (Fig. 2(c)) shows three CN bands, with band heads located around 358, 388, and 420 nm. The CN molecular bands originate from the recombination of carbon and nitrogen atoms in the plasma. The carbon atoms are from the cellulose fiber of the filter paper and the nitrogen atoms are from the surrounding air. Along with the CN bands, atomic lines of Mg I, Mg II, Na I, Ca I, Ca II, Fe I, Al II, Sr II and Ba II were also found. These atomic and ionic emission lines originated from the ash content of the FP. The spectrum of the 11-element sample with FP as substrate is shown in Fig. 2(d). Apart from the lines shown in Fig. 2(c), atomic emission lines of all these 11 testing elements can be found in Fig. 2(d), including Au I (328.068 nm), Cr I (357.869, 425.435 and 428.972 nm), Cu I (324.754, 327.396 nm), Eu II (381.967, 412.970 and 420.505 nm), In I (451.131 nm), Mn I (279.482, 403.076 and 403.307 nm), Ni I (300.249, 341.476 and 352.454 nm), Rh I (343.489, 369.236 and 365.799 nm), Y II (324.228, 360.073, 371.030 and 377.433 nm), Sr II (407.771 nm) and Ba II (455.403 nm). Therefore, we can make a conclusion that FP substrate was necessary for the detection of these elements through surface sampling — no FP, no signal. With FP as substrate, the sample spot is subject to both radiation heating by the plasma jet and the combustion heating by burning FP during ablation. The combustion of the FP made up for the energy deficiency of the plasma jet, which effectively promoted the atomization/excitation processes.

**Figure 3:**
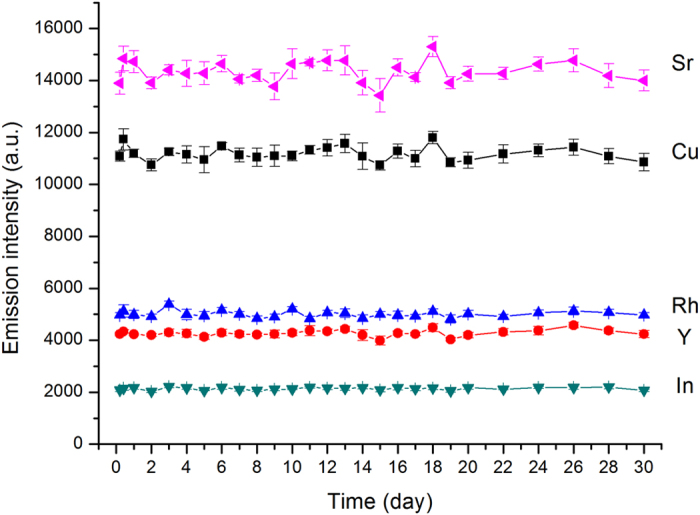
Stability of emission intensity of Cu (324.754 nm), Rh (343.489 nm), Y (371.030 nm), Ba (455.403 nm) and In (451.131 nm). Error bars in the figure represent standard deviations of the results (n = 10). The concentrations of Cu, Rh, Y and In were 4 μg mL^−1^, Sr was 0.8 μg mL^−1^, with sample volume of 1 μL.

The small precut FP matrix also helps to maintain the sample amount in unit surface and is suitable for sample storage. After sample being deposited on the FP, it can be detected immediately or allowed to dry (at least four hours) for storage. In the latter case, these dried sample FPs can be stacked and stored in resealable plastic bags or plastic containers. The stability of analytes in dried sample spots was evaluated by exposing samples (4 ng Cu, Rh, Y, In and 0.8 ng Ba) sealed in plastic bags at room temperature (22–28 °C) for up to 30 days. As shown in Fig. 3, the emission intensities of all the five nonvolatile elements were very stable. The FP matrix appears to provide some protection against sample degradation. As a result, FP can be considered as an ideal method for keeping sample stable over time and facilitating transportation of samples.

### The blank correction process

**Figure 4:**
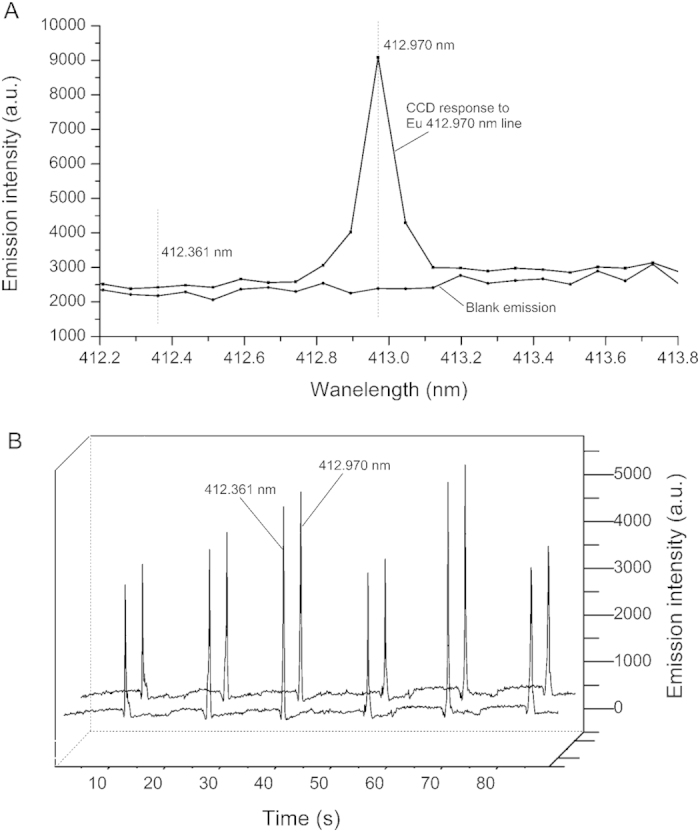
(**a**) The CCD responses to the 412.970 nm Eu atomic line and blank emission. (**b**) Temporal emission profiles of blank emission at 412.970 nm and 412.361 nm for 6 consecutive measurements.

**Figure 5:**
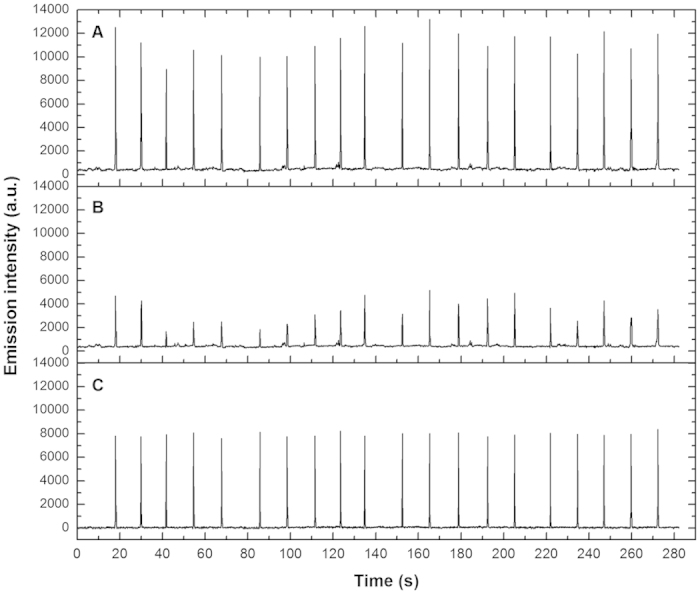
Blank correction for the emission intensity of Eu at 412.970 nm with respect to the blank emission obtained at 412.361 nm. (**a**) The emission intensity recorded at 412.970 nm for 5 μg mL^−1^ Eu; (**b**) the blank emission; (**c**) the net emission intensity after performing blank correction.

Combustion of FP inevitably gives out lots of visible light, and the instability of combustion contributes to a source of variation, resulting in remarkable elevation of spectral baseline in the visible region and large fluctuations in the emission of blank. This variation significantly deteriorates the accuracy and precision of the entire analytical process. Emission intensity at peak position can’t be regarded as peak height for quantitative determination due to the irregular undulations of baseline. Therefore, blank correction is of great importance in quantitative analysis. Since Eu has a secondary sensitive line 412.970 nm located in the visible region, it was selected as an example for testing. Figure 4(a) shows the emission spectra of 4 μg mL^−1^ Eu and blank emission in the region between 412.2 to 413.8 nm. The wavelength of 412.361 nm is adjacent to the peak area of Eu 412.970 nm. The temporal signal profiles of blank emission at 412.970 nm and 412.361 nm were recorded simultaneously for six measurements and the results are shown in Fig. 4(b). It can be found that fluctuations of blank emissions at the two neighboring wavelengths (412.970 nm and 412.361 nm) follow exactly a same trend during whole measurement cycles. And the signal at the two wavelengths have similar intensities, the emission intensity at 412.361 nm is around 95% of that at 412.970 nm. Therefore, the emission intensity at 412.361 nm can be regarded as the blank data for 412.970 nm. The blank correction can be performed by first recording the emission signals at both 412.970 nm and 412.361 nm (Fig. 5(a,b)), and then the net intensity of 412.970 nm atomic line can be derived by subtracting the corresponding blank data at 412.361 nm (Fig. 5(c)). As shown in Fig. 5(c), the blank correction process effectively eliminated the interferences of visible light caused by combustion of FP and significantly improved the reproducibility of analysis. Blank correction was done for every element quantitatively analyzed in this work. The analytical lines and the corresponding blank correction wavelengths for the 8 elements tested are listed in Table 1.

### Analytical performance and figures of merit

**Figure 6:**
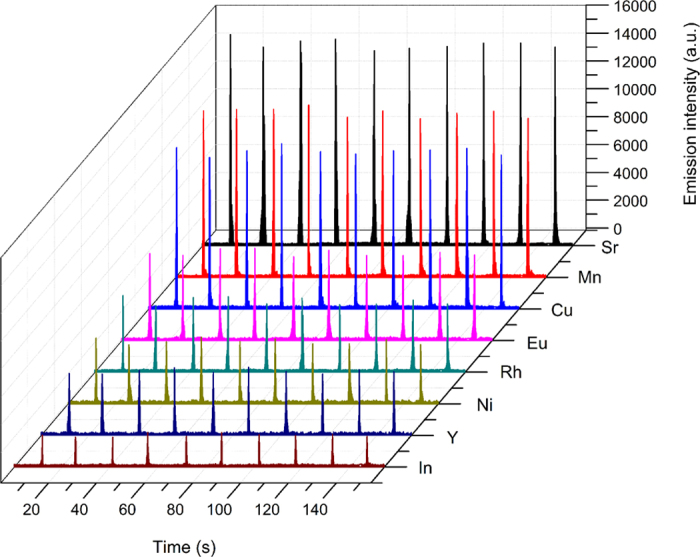
Temporal emission profiles of 10 consecutive determinations of In (451.131 nm), Y (371.030 nm), Ni (352.454 nm), Rh (343.489 nm), Eu (412.970 nm), Cu (324.754 nm), Mn (403.076) and Ba (455.403 nm). The concentrations of In, Y, Ni, Rh, Eu, Cu and Mn were 4 μg mL^−1^, Ba was 0.8 μg mL^−1^, with sample volume of 1 μL.

The analytical performance of our MAPAES technique was evaluated under optimal conditions (microwave power: 150 W, argon flow rate: 300 mL/min). The net emission peak was used as analytical signal throughout this work. Linear correlation coefficients (R) for calibration curves of the 8 analytes were all better than 0.99. Limits of detection (LOD) were calculated using the definition 3 s/m, where s is the standard deviation of 11 blank measurements and m is the slope of the calibration plot. The LOD values of the 8 elements range from 1.6 to 59 ng mL^−1^ corresponding to absolute mass LODs of 1.6 to 59 pg in 1 μL sample solution. The detection limits of the proposed MAPAES system have also been compared with other OES systems (cf. Table 1). It can be seen that, with little sample consumption, the detection limits for this new source are comparable to or one order higher than those of ICPAES and MIPAES. In addition, repeatability, expressed as relative standard deviation from 10 replicates, ranged from 2.5% to 5.6% for analyte concentrations at 0.8 μg mL^−1^ (Ba) and 4 μg mL^−1^ (others). Figure 6 shows the temporal profile of emission signal from 10 consecutive determinations of an 8-element standard solution. These results indicate that this new system has acceptable sensitivity and repeatability.

To validate the proposed method, the MAPAES system was employed to determine Cu, Mn, Ba and Ni in standard reference materials of simulated natural water samples (GBW(E)080397, GBW(E)080406, and GBW(E)080405) and a real water sample (river water). In comparison with conventional ICPAES and MIPAES, MAPAES requires less sample pretreatment. Pneumatic nebulizers as the most common sample introduction device for ICP and MIP, can only sampling clean solution without any precipitate. However, MAPAES is able to analysis turbid liquid samples directly. The real water sample can be added onto the sample plate for direct analysis without filtration or acidification. The analytical results of each element in these standard reference materials and the water sample are summarized in Tables 2 and 3. The analytical results obtained with the proposed method are well in agreement with the certified values. In addition, real sample recovery was also tested with spiked 500 ng mL^−1^ of Cu, Mn, Ba and Ni (97.6–102.4%), and satisfactory results were obtained, confirming that the proposed method is valid for analysis of environmental water samples.

## Discussion

A novel method for surface sampling elemental analysis was developed based on the matrix-assisted plasma atomization emission spectrometry. With the assistance of filter paper, multiple elements on its surface can be detected directly by the plasma tail plume with good sensitivity. It can be concluded that the FP matrix provides a number of essential functions: (1) offering energy to assist the atomization/excitation process by combusting itself, (2) as an absorbing matrix for analytes, preventing the sample solution/powder diffusion or splash, (3) without complex sample pretreatment, (4) possibly direct detection of elements using plasma tail plume through surface sampling, (5) stabilizing analytes in dried sample spots at room temperature, thus providing considerable flexibility for sample collection and transportation.

The present method provides a new way for atomic spectrometry and offers several advantages. First, MAPAES enables simultaneously fast analysis (less than 1 min per sample) for multiple elements without any sample pretreatment, which is promising for high-throughput analysis. Second, analysis by MAPAES consumes only low sample volume (1 μL), a benefit for samples with limited amount. In addition, MAPAES eliminates the use of a sample flow system. The whole system is small in size, simple in structure, and cost-efficient, which provides a possibility to design a portable, miniature MAPAES instrument for field analysis.

## Methods

### The MAPAES system

The home-built microwave-induced plasma source was in a Surfatron structure, which employed surface wave for energy propagation. The microwave coaxial cavity was in a cylinder-shape and made of copper with a fused-silica tube (1.0 mm i.d., 6 mm o.d., 200 mm long) centered axially. The rf power from the solid-state microwave generator (2450 MHz, maximum power of 150 W) was input into the cavity via a standard N-type connector. Both inner chamber length and gap length of Surfatron were adjustable to get the highest microwave coupling efficiency. By easily rubbing the internal surface of the fused-silica tube with a slender metal wire, discharge was started and a stable, cone-shaped plasma jet was generated. The stability of the microwave induced plasma was examined over a period of 6 hours. As can be seen from Figure SI-1, there is very limited fluctuation in background signals during this time period.

Commercial quantitative filter paper (ashless grades, grade 44, Whatman, Φ70 mm) was used as the sample substrate after being cut into small pieces (2.5 mm × 1.5 mm, L × W). Alumina ceramic chip (50 mm × 20 mm × 1 mm, L × W × T) was chosen as the baseplate due to its high heat resistance (>1000 °C) and opaqueness. Sample plate was fabricated by pasting 10 pieces of the precut FP into two columns on the ceramic baseplate by homemade PVA glue (Fig. 1(c)).

The microwave cavity was mounted onto a vertical rotating stage, which allowed selection of impact angles of 0° to 90°. The argon stream, controlled by a mass flow controller (D07-19B, Beijing Sevenstar Electronics CO., LTD), was fed through the fused-silica tube at a flow rate of 0.2–2.0 L min^−1^. The length of plasma jet extending beyond the fused-silica tube was 2–10 mm. For analysis, the sample plate was placed on a goniometer stage, which was mounted on a 3D translational stage to allow precise alignment of the plasma jet with the sample column. The analyte was detected *in situ* on the surface of the sample plate. By manually translating the ceramic baseplate, the analyte contained FP was ablated by plasma tail plume successively. The light emitted from the atomic emission process was picked up and focused into an optical fiber by a 5 mm diameter collimate lens and the emission spectra were recorded by an Avaspec four channel fiber optic spectrometer system (AvaSpec-2048-4-DT). The liner CCD-array detector with 2048-element pixels was able to simultaneously detect the whole spectrum from 200 to 825 nm. An integration time of 40 ms, an average of two scans were set for the CCD to ensure a reasonable signal-to-noise ratio. This spectrometer has 8 functions for signal collection, which enables simultaneously quantitative analysis of at most 8 elements.

### Reagents and standards

All reagents used in this work were of at least analytical grade. Ultrapurified water (18.2 MΩ cm^−3^) obtained from a UP water purification system was used throughout this work. Stock solutions (1000 mg L^−1^) of Au, Ba, Cr, Cu, Eu, In, Mn, Ni, Rh, Sr, and Y were purchased from the National Research Center for Standard Materials (NRCSM) of China. Working standards were prepared from diluting stocking solution with nitric acid (5%).The discharge gas, high-purity argon (99.99%) was provided by Qiao Yuan Gas Company. Reference materials, including simulated natural water samples GBW(E)080397, GBW(E)080406, and GBW(E)080405, were used to validate the accuracy of our method. A water sample was collected from Funan River in Chengdu city.

### Safety considerations

Electrical shock may happen on igniting the discharge with a slender metal wire. Extreme care and precaution should be taken to prevent electrical shock through the use of electrically insulating gloves. Personal protective equipment, such as microwave protective clothing and safety glasses, are highly recommended to prevent microwave radiation.

## Additional Information

**How to cite this article**: Yuan, X. *et al*. Matrix-Assisted Plasma Atomization Emission Spectrometry for Surface Sampling Elemental Analysis. *Sci. Rep.* **6**, 19417; doi: 10.1038/srep19417 (2016).

## Supplementary Material

Supplementary Information

## Figures and Tables

**Table 1:** Analytical characteristics of elemental determination with MAPAES system and compared with other optical emission spectrometric systems.

element	wavelength(nm)	blank correctionwavelength(nm)	present workRSD(%)	present workLOD(ng mL^−1^)	present workLOD (pg)	present worklinear range(μg mL^−1^)	ICP-AESLOD (ng mL^−1^)	MIP-AESLOD (ng mL^−1^)	APGD LOD(ng mL^−1^)	ELCAD LOD(ng mL^−1^)
Ba	455.403	454.384	4.1	1.6	1.6	0.02–1	8^6^	41^33^	–	6.9^34^
Cu	324.754	324.993	2.5	7.6	7.6	0.05–5	1.5^35^	2.1^36^	77^37^	31^38^
Eu	412.970	412.361	4.1	19	19	0.1–10	0.34^39^	–	–	–
In	451.131	450.835	5.5	59	59	0.5–30	–	–	40^40^	–
Mn	403.076	402.844	2.7	11	11	0.05–5	1^6^	9.2^33^	100^41^	30^38^
Ni	352.454	352.679	5.6	41	41	0.2–20	65^6^	6.2^36^	1800^37^	11^38^
Rh	343.489	343.785	5.6	15	15	0.1–15	120^42^	1.8^43^	–	–
Y	371.030	370.866	4.6	42	42	0.5–20	3.4^44^	–	–	–

**Table 2:** Determination of different elements in the certified reference materials by the present system.

sample	element	certified value(μg mL^−1^)	measured value(μg mL^−1^)
GBW(E)080397	Cu	10.00 ± 0.20	9.96 ± 0.08
GBW(E)080406	Mn	5.00 ± 0.10	4.85 ± 0.14
GBW(E)080405	Ni	5.00 ± 0.20	5.02 ± 0.15

**Table 3:** Analytical results for four elements (Cu, Ba, Mn and Ni) in river water sample.

sample	element	added (ng mL^−1^)	detected^a^ (ng mL^−1^)	recovery (%)
River water	Cu	0	nd	–
		500	488 ± 12	97.6
	Ba	0	nd	–
		500	512 ± 22	102.4
	Mn	0	58	–
		500	549 ± 30	98.2
	Ni	0	nd	–
		500	505 ± 13	101.0

^a^Mean ± SD (n = 10); nd: not detectable.
