# Supplementary material for: Matrix-Assisted Plasma Atomization Emission Spectrometry for Surface Sampling Elemental Analysis
Source: Sci Rep. 2016 Jan 14;6:19417. doi: 10.1038/srep19417 (PMC4725919; doi:10.1038/srep19417)
Supplement: Supplementary Information [file srep19417-s1.doc]

**Supporting Information**

**Matrix-Assisted Plasma Atomization Emission Spectrometry for Surface Sampling Elemental Analysis**

Xin Yuan^a^, Xuefang Zhan^b^, Xuemei Li^b^, Zhongjun Zhao^a^ and Yixiang Duan^b^*

^a^ Research Center of Analytical Instrumentation, College of Chemistry, Sichuan University, Chengdu, China, 610064

^b^ Research Center of Analytical Instrumentation, Key Laboratory of Bio-resource and Eco-environment, Ministry of Education, College of Life Science, Sichuan University, Chengdu, China, 610064

* To whom correspondence should be addressed. Phone: +86-28-85418180. Fax: +86-28-85418180. Email: [yduan@scu.edu.cn](mailto:yduan@scu.edu.cn)

1. **Plasma stability**

The stability of the microwave induced plasma over a period of 6 hours was examined by measuring the background emission intensity at 301.284 nm, N_2_ at 380.495 nm and 405.818 nm. The signals were recorded every 5 min. Figure SI-1 shows the change of emission intensity versus operating time. There is very limited fluctuation in background and N_2_ signals. The relative standard deviation (RSD) for the emission intensity was 2.4% for background, 2.7% for N_2_ (405.818 nm), and 3.4% for N_2_ (380.495 nm).


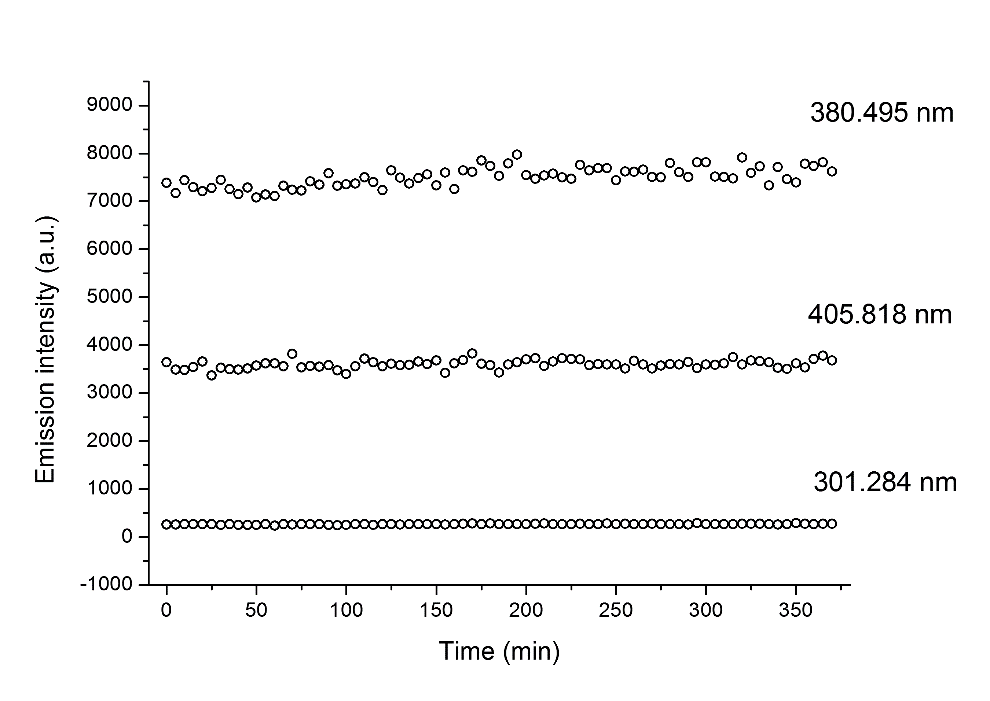


**Figure SI-1. Stability of the microwave induced plasma source:** Ar flow rate, 300 mL min^-1^; microwave power, 150 W; CCD integration time, 40 ms.

1. **Effect of pH**

The influence of pH on the net-emission intensity of 4 μg mL^-1^ Cu, Rh, Y, In and 0.8 μg mL^-1^ Ba was investigated. Solution pH value from 0 to 7 was examined, and the results are presented in Figure SI-2. The net-emission intensity of all the five elements kept stable with the pH value varies from 0 to 7. Therefore, we can conclude that pH has little influence on signal intensities. This is because in the carbonization process, the sample solution (together with volatile acid) was completely evaporated due to the thermal effect of the plasma. As a result, pH adjustment was unnecessary in this work and standards or samples were directly analyzed after dilution with DIW.


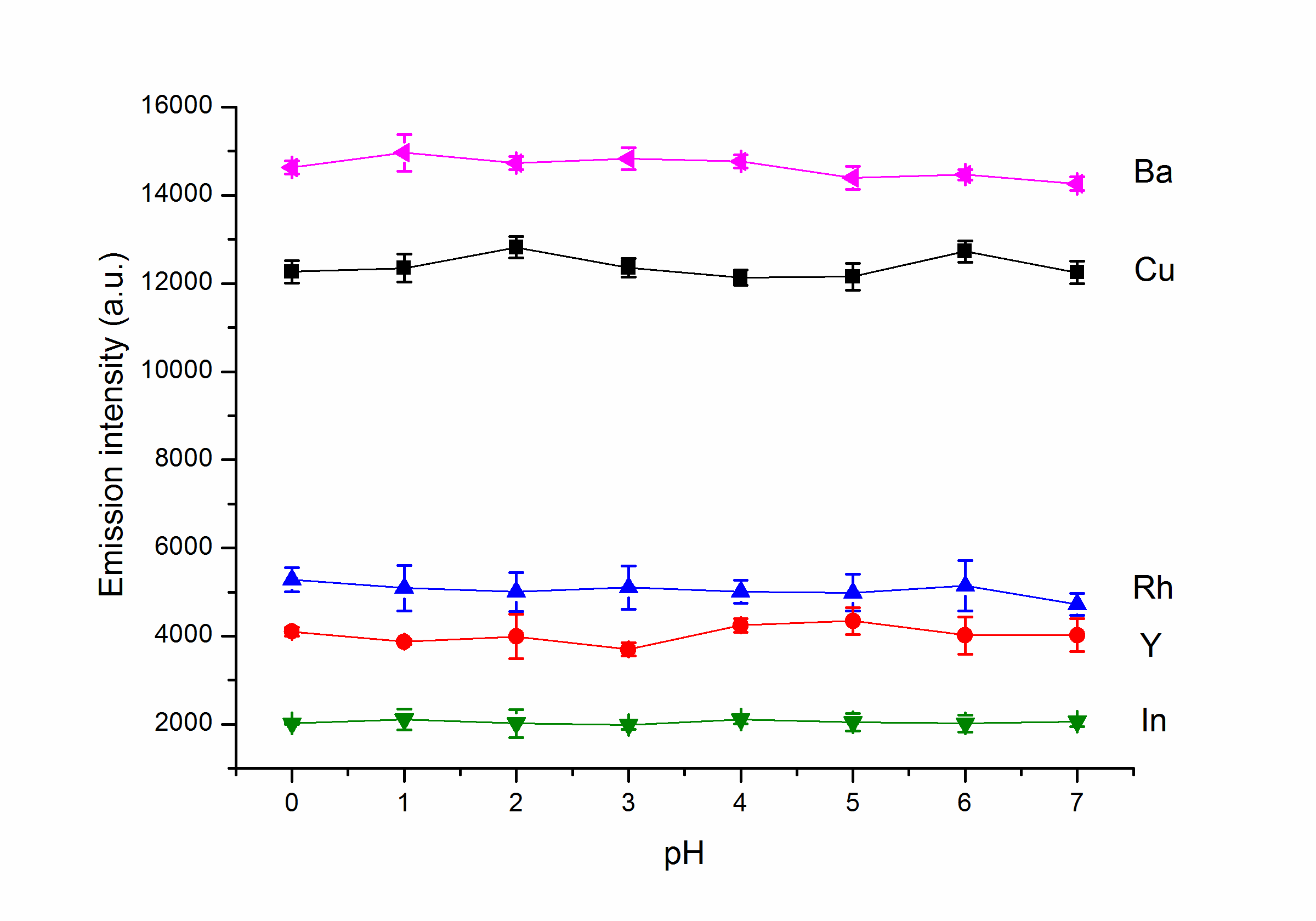


**Figure SI-2 Effect of pH on net-emission intensity of Ba (455.403 nm), Cu (324.754 nm), Rh (343.489 nm), Y (371.030 nm) and In (451.131 nm).** Error bars in the figure represent standard deviations of the results (n=10). The concentrations of Cu, Rh, Y and In were 4 μg mL^-1^, Ba was 0.8 μg mL^-1^, with sample volume of 1 μL.
